# 4MS AGE-FRIENDLY HEALTHCARE INITIATIVE IMPLEMENTATION ON AN ACUTE CARE FOR ELDERS UNIT

**DOI:** 10.1093/geroni/igac059.2021

**Published:** 2022-12-20

**Authors:** Lucy Wilson, Chelsea Rick, Sarah Welch, Sarah Nelson, Olivia Lawson, Hannah Boren, Mariu Duggan

**Affiliations:** Vanderbilt University Medical Center, Nashville, Tennessee, United States; Vanderbilt University Medical Center, Nashville, Tennessee, United States; Vanderbilt University Medical Center, Nashville, Tennessee, United States; Vanderbilt University Medical Center, Nashville, Tennessee, United States; Vanderbilt University Medical Center, Nashville, Tennessee, United States; Vanderbilt University Medical Center, Nashville, Tennessee, United States; Vanderbilt University Medical Center, Nashville, Tennessee, United States

## Abstract

Although evidence supports 4Ms [Medication, Mentation, What Matters Most (WMM), Mobility] care for older adults, successful implementation at an academic hospital is not well described. The aim of this study was to describe the implementation methods and process measures for 4Ms delivery on the Acute Care for Elders (ACE) unit at an academic hospital. We used the Institute for Healthcare Improvement’s Model for Improvement to guide efforts. We set SMART goals for each M: best possible medication reconciliation, identification of potentially inappropriate medications, and recommendations to deprescribe (medication), delirium screen documented 2x/day and delirium nonpharmacologic protocol in place (mentation), documentation of WMM and care alignment (WMM), Johns Hopkins-Highest Level of Mobility screen assessed during the patient’s hospitalization, mobilization of patient 3x/day offered and documented, and restraints avoided (mobility). We mapped current and ideal workflows. We sought community grant funding to expand the implementation team, supporting a nurse educator to train the unit and data analyst to extract real-time data from the electronic medical record to inform improvement processes. Multiple Plan-Do-Study-Act cycles were run iteratively and discussed at weekly team meetings. We included patients >65 years old, admitted for >48 hours, and excluded patients admitted on hospice. Of 519 eligible patients admitted from 04/2021-01/2022, goals were met by 454 (87%) for medication, 187 (36%) for mentation, 130 (25%) for WMM, and 6 (1%) for mobility. We found implementing 4M care processes at an academic hospital to be feasible. Further exploration of barriers to meeting the mobility target is warranted.

